# Novel Facets of the Liver Transcriptome Are Associated with the Susceptibility and Resistance to Lipid-Related Metabolic Disorders in Periparturient Holstein Cows

**DOI:** 10.3390/ani11092558

**Published:** 2021-08-31

**Authors:** Ryan S. Pralle, Wenli Li, Brianna N. Murphy, Henry T. Holdorf, Heather M. White

**Affiliations:** 1 Department of Animal and Dairy Sciences, University of Wisconsin-Madison, Madison, WI 53706, USA; praller@uwplatt.edu (R.S.P.); hholdorf@wisc.edu (H.T.H.); 2 School of Agriculture, University of Wisconsin-Platteville, Platteville, WI 53818, USA; 3 Dairy Forage Research Center, USDA-Agricultural Research Service, Madison, WI 53706, USA; bmurphy8@wisc.edu

**Keywords:** dairy cow, transition period, ketosis, fatty liver, RNA-Seq, clustering, liver metabolism

## Abstract

**Simple Summary:**

Energy and nutrient demands of the early lactation period can result in the development of metabolic disorders, such as ketosis and fatty liver, in dairy cows. Variability in the incidence of these disorders suggests that some cows have an ability to adapt. The objective of this study was to discover differences in liver gene expression that are associated with a cow’s susceptibility (disposition to disorder during typical conditions) or resistance (disposition to disorder onset and severity when presented a challenge) to metabolic disorders. Cows in a control treatment and a ketosis induction protocol treatment were retrospectively grouped into susceptibility and resistance groups, respectively, by a machine learning algorithm using lipid biomarker concentrations. Whole-transcriptome RNA sequencing was performed on liver samples from these cows. More susceptible cows had lower expression of glutathione metabolism genes, while less resistant cows had greater expression of eicosanoid-metabolism-related genes. Additionally, differentially expressed genes suggested a role for immune-response-related genes in conferring susceptibility and resistance to metabolic disorders. The overall inferred metabolism suggests that responses to oxidative stress may determine susceptibility and resistance to metabolic disorders, with novel implications for immunometabolism.

**Abstract:**

Lipid-related metabolic disorders (LRMD) are prevalent in early lactation dairy cows, and have detrimental effects on productivity and health. Our objectives were to identify cows resistant or susceptible to LRMD using a ketosis induction protocol (KIP) to discover differentially expressed liver genes and metabolic pathways associated with disposition. Clustering cows based on postpartum lipid metabolite concentrations within dietary treatments identified cows more or less susceptible (MS vs. LS) to LRMD within the control treatment, and more or less resistant (MR vs. LR) within the KIP treatment. Whole-transcriptome RNA sequencing was performed on liver samples (−28, +1, and +14 days relative to calving) to assess differential gene and pathway expression (LS vs. MS; MR vs. LR; *n* = 3 cows per cluster). Cows within the MS and LR clusters had evidence of greater blood serum β-hydroxybutyrate concentration and liver triglyceride content than the LS and MR clusters, respectively. The inferred metabolism of differentially expressed genes suggested a role of immune response (i.e., interferon-inducible proteins and major histocompatibility complex molecules). Additionally, unique roles for glutathione metabolism and eicosanoid metabolism in modulating susceptibility and resistance, respectively, were implicated. Overall, this research provides novel insight into the role of immunometabolism in LRMD pathology, and suggests the potential for unique control points for LRMD progression and severity.

## 1. Introduction

The physiological adaptations necessary to make the transition from a gravid, non-lactating state through parturition and into a lactating state represent several metabolic challenges for periparturient dairy cows. The principal challenges include onset of negative net energy and nutrient balance spurred by a reduction in voluntary feed intake and increasing lactation energy requirements, insulin resistance, immunosuppression, and mineral imbalance [1,2,3,4]. Responses to these challenges, such as the degree of body nutrient mobilization and hepatic nutrient metabolism, can vary [5,6,7,8]. Maladaptation to these challenges can result in numerous pathologies, including the lipid-related metabolic disorders (LRMD) hyperketonemia (HYK) and fatty liver syndrome (FLS). These comorbid disorders are prevalent in early lactation dairy cows, with an incidence of 43–53% and 50% for HYK and FLS, respectively [9,10,11,12]. Hyperketonemia—defined as elevated concentrations of blood β-hydroxybutyrate (BHB)—and bovine FLS—characterized by a substantial accumulation of liver triglyceride (TG)—have been associated with unfavorable performance outcomes, including greater risk of comorbidities, decreased reproductive efficiency, productive losses, and premature culling [7,9,13,14,15]. The deterministic estimate for total cost per case of hyperketonemia is USD 375 and USD 256 for primiparous and multiparous cows, respectively [16]. The financial impact of fatty liver on dairy production is difficult to determine, but was previously expected to cost the industry at least USD 60 million annually [14]. Therefore, these LRMD represent a concern not only for the health and wellness of dairy cows, but also for the cost of dairy production.

Our understanding of the pathology of these disorders has historically focused on understanding the liver metabolism of long-chain fatty acids (FA) mobilized from adipose tissue TG [14,17]. An apparent excess of liver uptake of endogenous FA surpasses the oxidative and secretory capacity of hepatocytes, promoting ketogenesis and storage of re-esterified FA [14,17,18]. Thus, most research regarding the prevention and treatment of HYK and FLS has focused on limiting FA substrates by managing dairy cows’ prepartum obesity or excessive mobilization of FA during the postpartum period [19,20,21]. Alternative approaches have been focused on nutritional interventions to support oxidation via TCA [22,23,24] or to improve liver TG secretion [25,26,27].

The advent of genome-wide gene expression profiling using microarray or RNA-Seq techniques has expanded our capability to identify and understand the underpinning regulatory mechanisms of physiological states. With respect to the liver transcriptome, research concerning LRMD has focused on comparing prepartum dietary treatments (lower vs. greater dietary energy) [28,29,30,31] or feed restriction models [32,33] to either increase the risk of metabolic disorders or induce negative energy balance. In addition, the liver transcriptome has been compared between cows overfed dietary energy achieving subclinical HYK and those not progressing to HYK [34]. These papers have given insight into the pathogenesis of LRMD, such as highlighting the role of key transcription factors (i.e., peroxisome proliferator-activated receptor) and the contributions of steroid biosynthesis [28,29,30,31,32]. However, the liver transcriptome of periparturient dairy cows experiencing LRMD absent a challenge protocol has not been compared to apparently healthy controls. Additionally, there has been no evaluation of whether the differential biology observed during these experiments is representative of any differential biology in LRMD that occur without a challenge protocol.

We hypothesize that periparturient cows have divergent physiological adaptations to lactation that affect their susceptibility or resistance to LRMD. Furthermore, we hypothesize that there are unique genes and metabolic pathways responsible for LRMD susceptibility and resistance. Our presupposition is that dairy cows developing LRMD pathologies absent of a challenge represent an LRMD-susceptible population of cows, while cows with delayed onset or severity of LRMD when challenged represent a population of LRMD-resistant cows. To evaluate our hypotheses, we clustered periparturient cows based on postpartum lipid metabolite concentrations within a control treatment (CTL; no dietary challenge) and a ketosis induction protocol (KIP; dietary challenge with oversupply of energy prepartum and postpartum feed restriction). Within the CTL treatment, we identified a cluster of cows apparently less susceptible (LS) to LRMD and a cluster more susceptible (MS) to LRMD; additionally, we determined a cluster apparently more resistant (MR) to LRMD and a cluster less resistant (LR) to LRMD in the KIP treatment. Our objectives were to evaluate the liver transcriptome of these clusters via RNA sequencing within the original treatment for differentially expressed genes (DEGs) and enriched metabolic pathways (EMPs), as well as to evaluate the common and unique liver transcriptome features of the susceptibility and resistance models.

## 2. Materials and Methods

### 2.1. Animal Experimental Design

This research was part of a previously detailed experiment that enrolled multiparous Holstein cows (*n* = 25) at the University of Wisconsin–Madison Dairy Cattle Instruction and Research Center [35]. All animal use and handling protocols were approved by the University of Wisconsin–Madison College of Agricultural and Life Sciences’ Animal Care and Use Committee (protocol A005467-R01). Blocked by expected calving date, cows were randomly assigned to a CTL (*n* =13) or a KIP (*n* = 12) treatment. The experimental period began at −28 days relative to calving (DRTC) and ended at +56 DRTC. Control cows were allowed ad libitum intake of diets formulated to meet the needs of dry or lactating cows, respectively (Supplementary Table S1). The KIP cows were offered a daily top-dress of dry, cracked corn (6 kg) in addition to ad libitum access to the dry cow ration [35]. Post-calving, KIP cows were offered ad libitum access to the lactating cow ration until +14 DRTC, at which time feed intake was restricted to 80% of ad libitum intake, based on the average voluntary intake from the 3 days preceding restriction. Blood BHB was monitored daily for all postpartum cows with a BHBCheck meter (PortaCheck, Moorestown, NJ); cows that achieved a blood BHB ≥ 3.0 mmol/L were treated for clinical ketosis, re-alimented to feed, and allowed ad libitum intake for the remainder of the experiment. All KIP cows and 2 CTL cows achieved blood BHB ≥ 3.0 mmol/L [35]; treatment for clinical ketosis included intravenous dextrose (250 mL; Phoenix Scientific Inc., St. Joseph, MO, USA; 50% dextrose), orally administered Propylene Advantage (300 mL/d for 3 to 5 days; TechMix LLC, Stewart, MN), and a B-vitamin complex injected intramuscularly (20 mL; Sparhawk Laboratories, Inc., Lenexa, KS, USA). Additional cow illnesses monitored by the herd veterinarian included displaced abomasum (*n* = 1 LR cow), clinical hypocalcemia (*n* = 1 LR cow), mastitis (*n* = 1 LS cow at +53 DRTC), retained placenta (*n* = 0 cows), and metritis (*n* = 0 cows).

Methods regarding sample collection and analysis are detailed in depth in the companion manuscript [35]. Briefly, feed intake and milk yield data were recorded daily, and composition analysis was performed on monthly composites of feed samples and on milk samples collected weekly. Sampling occurred at −28, −14, +1, +14, +28, +42, and +56 DRTC for body weight (BW), body condition score (BCS), blood samples, and blind percutaneous liver biopsies (Supplementary Methods) [35]. Sample collection at +14 DRTC preceded feed restriction for the KIP treatment. Additional body weights and BCSs were evaluated at −7 and +7 DRTC, and additional blood samples were collected at −7, −5, −3, +3, +5, and +7 DRTC. Blood fractions (serum or plasma) were quantified for BHB and glucose, respectively, using Catachem VETSPEC reagents on the Catachem Well-T AutoAnalyzer (Catachem, Oxford, CT, USA). Plasma FA concentration and liver TG concentration were determined using colorimetric, enzymatic assays [22,35,36].

### 2.2. k-Means Clustering and Retrospective Selection

Cows assigned to the CTL treatment were absent of an additional dietary challenge, and were leveraged to identify cows LS or MS to LRMD. Meanwhile, cows assigned to the KIP treatment were dietarily challenged to induce LRMD and leveraged to identify cows MR or LR to LRMD. To avoid subjectively choosing cows representing differential LRMD susceptibility or resistance, a *k*-means clustering algorithm (R, version 3.5.2) was used to empirically group cows within their original dietary (CTL or KIP) treatment based on metabolic characteristics. Variables supplied to the algorithm included concentrations of plasma FA, serum BHB, and liver TG from +1, +14, and +28 DRTC, as well as the maximum postpartum concentration of each lipid metabolite from any DRTC timepoint. This allowed for a longitudinal assessment of LRMD status, and the maximum concentrations served as a proxy for LRMD severity. All variables were tested for normality (*p* ≤ 0.05, Shapiro–Wilk test) and transformed (log_10_ or reciprocal) to an empirically Gaussian distribution (*p* > 0.05, Shapiro–Wilk test). For each original treatment, we evaluated algorithms allowing for 2–5 clusters, 1000 iterations, and 1000 random starts. Based on silhouette plot evaluation (R package: cluster, version 2.1.0) [37] and the number of cows within the largest 2 clusters (*n* ≥ 4), the optimal number of clusters was 2 and 4 for CTL and KIP cows, respectively. Two of the clusters from within the KIP treatment had too few cows to be considered for RNA-Seq (*n* ≤ 2) and were excluded. From the clusters within CTL and KIP treatments, cows (*n* = 4 per cluster) were randomly selected to represent the cluster, and proceeded to RNA isolation and library preparation for RNA-Seq. Poor RNA integrity, detailed subsequently, resulted in the elimination of one cow per cluster; therefore, each cluster was represented by 3 randomly selected cows (*n* = 6 per original treatment) with adequate RNA integrity. Assignment of clusters as LS or MS in the CTL treatment and LR or MR in the KIP treatment was based on the statistical evaluation of the metabolites supplied for clustering (see Statistical Analysis). Greater concentrations of LRMD biomarkers suggest progressed pathology and increased risk of negative performance outcomes [14,17,38,39]; therefore, the clusters with greater blood BHB, blood FA, or liver TG were designated MS and LR within the CTL and KIP treatments, respectively, and clusters with lower lipid metabolites were designated LS and MR within the CTL and KIP treatments, respectively. Parity distribution across clusters was 8 second parity, 2 third parity (MS cluster), 1 fourth parity (LR cluster), and 1 fifth parity (LS cluster).

### 2.3. RNA Isolation, Library Preparation, Sequencing, and Mapping

Liver tissue samples (~50 mg) were homogenized into fine powders in liquid nitrogen using a mortar and pestle. Sample RNA was extracted following the miRNeasy protocol with a QIAcube instrument (Qiagen, Foster City, CA, USA). The RNA integrity number was determined via Bioanalyzer using the RNA 6000 nano kit (Agilent, Santa Clara, CA, USA) for all samples (*n* = 48). To improve overall RNA integrity for the experiment, one cow per cluster was dropped due to excessively low integrity for at least one DRTC. For the remaining samples (*n* = 36), RNA integrity was 6.0 ± 1.2 (SD). Library preparation for RNA sequencing was done using the Illumina TruSeq Ribo-Zero gold kit (Illumina, San Diego, CA, USA) following the manufacturer’s instructions. For each sample, 1 μg of total RNA was used as the input. The fragment distribution of prepared libraries was assessed via Bioanalyzer using the DNA 1000 kit (Agilent, Santa Clara, CA, USA). Quantification of prepared libraries was performed using a Kapa quantification kit (Kapa biosystems, Darmstadt, Germany) with a QuantStudio5 quantitative PCR instrument (Thermo Fisher, Waltham, MA, USA). Libraries were further normalized to ensure equal quantities before sequencing. Normalized, pooled samples were sequenced on an Illumina NextSeq 500 instrument (Illumina, San Diego, CA, USA) to obtain paired-end, 2 × 75 bp reads using a 150 high-output kit. Quality of reads was assessed via FastQC (https://www.bioinformatics.babraham.ac.uk/projects/fastqc/ accessed on 28 December 2019). Before sequence alignment, raw reads were filtered to remove those shorter than 50 bp. The mean number of paired-end reads per library was 34,137,191 ± 646,323 (SEM). The ribosomal depletion of sample libraries coupled with the deep read count suggests that our results will be comparable to poly-A-enriched preparation methods, despite the relatively low library RIN values [40]. For alignment, the genome reference and annotation files for *Bos taurus* (release 106, ARS-UCD 1.2) were downloaded from the NCBI (https://www.ncbi.nlm.nih.gov/assembly/GCF_002263795.1 accessed on 14 March 2020) for use. Raw reads from the whole transcriptome RNA-Seq libraries were aligned to the *Bos taurus* reference genome using STAR (2.5.2b). Read count quantification was done using cufflinks [41] with a sorted bam file generated by STAR as the input file. The expression levels of mRNAs in each sample were normalized to fragments per kilobase of transcript per million mapped reads (FPKM) by cufflinks [41].

### 2.4. Statistical Analysis

Due to the dependence of the retrospective cluster assignments on the original dietary treatment (CTL or KIP), the complete data (*n* = 12, selected cows) were divided into datasets based on original dietary treatment; therefore, all statistical analyses compared either LS vs. MS or LR vs. MR.

Analysis of biometric (i.e., BCS), productive (i.e., milk energy yield), and metabolite data (i.e., serum BHB) was performed using the SAS (version 9.4; SAS Institute Inc., Cary, NC) procedures UNIVARIATE and GLIMMIX. Several response variables had non-Gaussian distributions based on the Shapiro–Wilk test (*p* < 0.05). For those responses, data transformations were systematically evaluated, and transformations providing Gaussian distributions were selected either empirically, by Shapiro–Wilk test (*p* > 0.05), or subjectively, by histogram visualization (when empirical solutions were not found). The bimodal nature of calculated net energy balance necessitated downstream analysis to be performed on prepartum and postpartum timepoints separately. Linear mixed models were used to evaluate responses for evidence of differences between clusters (LS vs. MS or LR vs. MR, *n* = 3 cows/cluster), using the systematic model-building procedure [35]. The typical fixed effects included cluster, time, and cluster × time; the random effects included cow, cow nested within week of lactation (models with subsampling), and repeated measures of cow across time (when applicable). All models were assessed for improvement by additional covariates (i.e., previous lactation, 305 d mature equivalent milk yield, parity, and −28 DRTC measurement), controlling for heterogeneous variance, and the use of alternative variance–covariance structures (variance components were default) [35]. Preplanned contrasts were performed to compare clusters (LS vs. MS and MR vs. LR) across postpartum timepoints (+1 to +56 DRTC), and were reported when they provided new information with respect to the LMM. Fixed effects with *p* ≤ 0.05 were considered to have significant evidence for differences, while effects with 0.05 < *p* ≤ 0.10 were considered to have marginal evidence for differences. Whenever a cluster × time effect had some evidence for a difference (*p* ≤ 0.10), we made simple-effect comparisons of treatments within timepoints and corrected for multiplicity using the Bonferroni method. Treatment means are expressed as least squares means, and the 95% confidence intervals denoted as (lower limit, upper limit).

Differential gene expression analysis was done using the Cuffdiff function of Cufflinks [41] within each DRTC for LS vs. MS and MR vs. LR. *P*-values were corrected for multiplicity by false discovery rate [42], and are hereafter referred to as *Q*-values. The Database for Annotation, Visualization, and Integrated Discovery (DAVID) web-based software was used to evaluate gene ontologies and EMPs [43,44] for DEGs within a DRTC comparison. Genes supplied to the test list (termed gene list by DAVID) had *Q* ≤ 0.10 within the respective DRTC timepoint comparison of clusters. Instead of comparing a gene list to all genes in the *Bos taurus* genome, a customized background list was supplied to DAVID for the respective DRTC timepoint comparison of clusters. These customized background lists included all sequenced genes successfully tested within the respective DRTC comparison. Fisher’s exact statistics were extracted and corrected for multiplicity by false discovery rate [42]. Our evidence criteria for DEGs, gene ontologies, and EMPs were *Q* ≤ 0.05 and 0.05 < *Q* ≤ 0.10 for significant and marginal evidence, respectively.

## 3. Results

### 3.1. Phenotypic Characterization of Clusters

**Figure 1:**
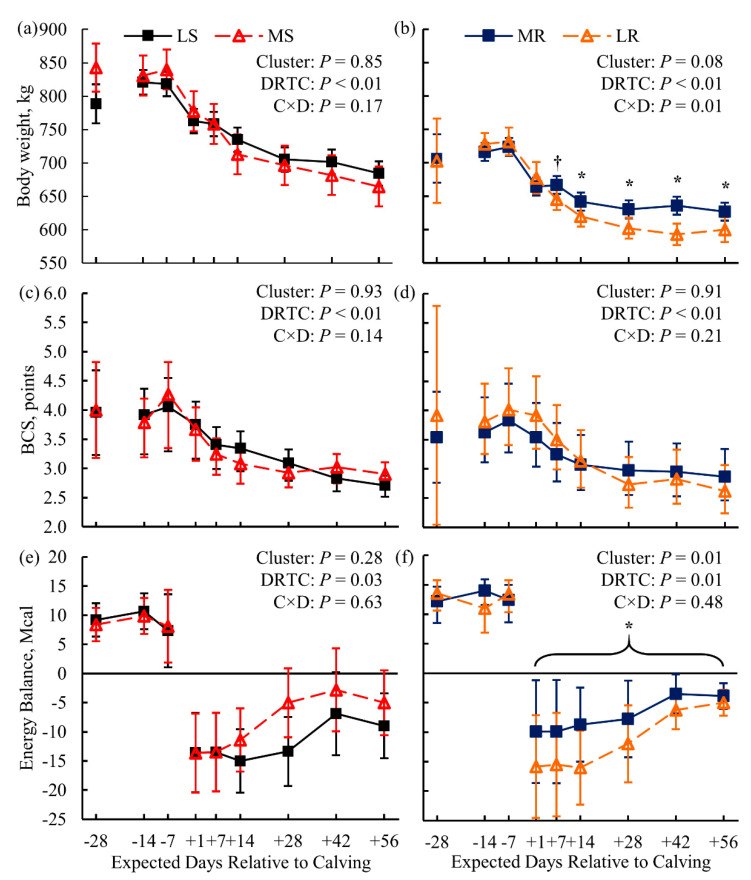
Body weight (panels **a** and **b**), body condition score (BCS; panels **c** and **d**), and calculated energy balance (panels **e** and **f**) for dairy cows clustered based on postpartum lipid metabolites within the original dietary treatment. Left-hand panels compare cows less (LS) or (MS) susceptible to lipid-related metabolic disorders, while right-hand panels compare cows more (MR) or less (LR) resistant to lipid-related metabolic disorders (*n* = 3 cows per cluster). Data are presented as least squares means (points) and their 95% confidence intervals (error bars); the exceptions are the arithmetic mean and 95% confidence interval for the −28 DRTC timepoint covariates (panels **a**–**d**). Statistics for the fixed effects of cluster, day relative to calving (DRTC), and their interaction (C × D) across the experimental period (panels **a**–**d**) or the postpartum period (panels **e** and **f**) are displayed in the top-right corner of each panel. Significant (*, *p* ≤ 0.05; Bonferroni adjusted) or marginal evidence (†, *p* = 0.07; Bonferroni adjusted) for the effects of cluster within DRTC or contrast of cluster across postpartum samples (bracketed) are indicated within their respective panels.

**Figure 2:**
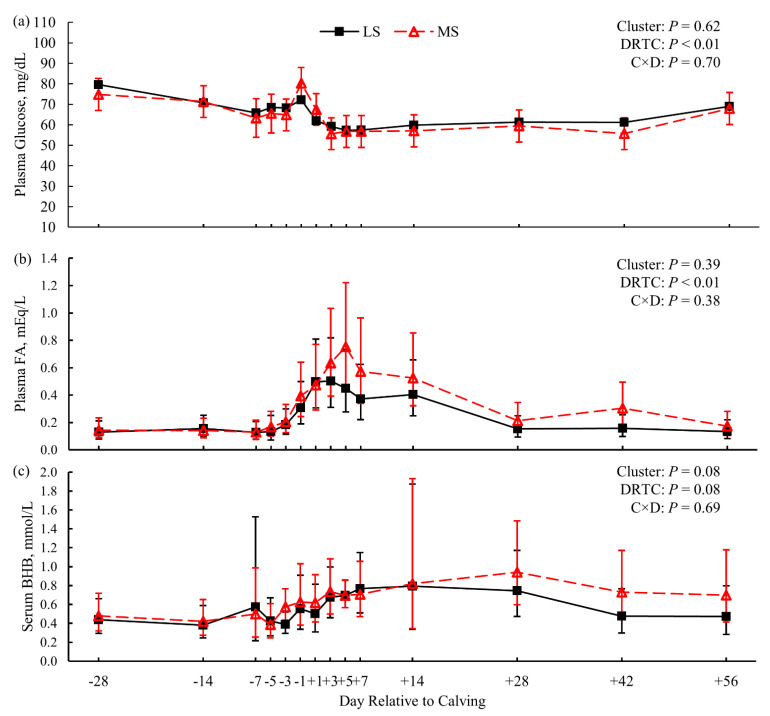
Blood fraction concentrations of glucose (panel **a**), fatty acids (FA; panel **b**), and β-hydroxybutyrate (BHB; panel **c**) for dairy cows less (LS) or more (MS) susceptible to lipid-related metabolic disorders (*n* = 3 cows per cluster). Data are presented as least squares means (points) and their 95% confidence intervals (error bars). Statistics for the fixed effects of cluster, day relative to calving (DRTC), and their interaction (C × D) are displayed in the top-right corner of each panel. Contrasts of cluster across postpartum samples did not reveal additional evidence of differences (*p* > 0.10; panels **a** and **b**).

**Figure 3:**
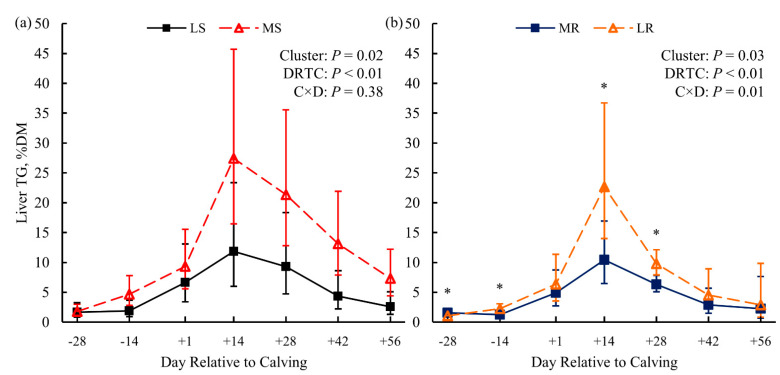
Liver triglyceride (TG) content for dairy cows clustered based on postpartum lipid metabolites within the original dietary treatment. Panel (**a**) depicts cows less (LS) or more (MS) susceptible to lipid-related metabolic disorders, while panel (**b**) compares cows more (MR) or less (LR) resistant to lipid-related metabolic disorders (*n* = 3 cows per cluster). Data are presented as least squares means (points) and their 95% confidence intervals (error bars). Statistics for the fixed effects of cluster, day relative to calving (DRTC), and their interaction (C × D) are displayed in the top-right corner of each panel. Significant evidence (*, *p* ≤ 0.02; Bonferroni adjusted) for simple effects of cluster within DRTC are indicated in their respective panels.

Biometric indicators of obesity—BW and BCS—were not different between LS and MS cows (*p* ≥ 0.85; Figure 1a,c). Milk energy output and dry matter intake (DMI) did not differ between susceptibility clusters (*p* = 0.59 and *p* = 0.41, respectively; Supplementary Table S2). Thus, net energy balance did not differ between susceptibility clusters pre- (*p* = 0.89; Supplementary Table S2) or postpartum (*p* = 0.28; Figure 1e). Plasma glucose (Figure 2a) and FA concentration (Figure 2b) did not differ (*p* = 0.62 and *p* = 0.39, respectively) between LS and MS cows either. Serum BHB concentration showed marginal evidence of being greater (*p* = 0.08) for the MS cows than for the LS cows (Figure 2c); a single MS cow was diagnosed with clinical ketosis (blood BHB ≥ 3.0 mmol/L). In addition, liver TG content was greater (*p* = 0.02; Figure 3a) for the MS cows than for the LS cows.

**Figure 4:**
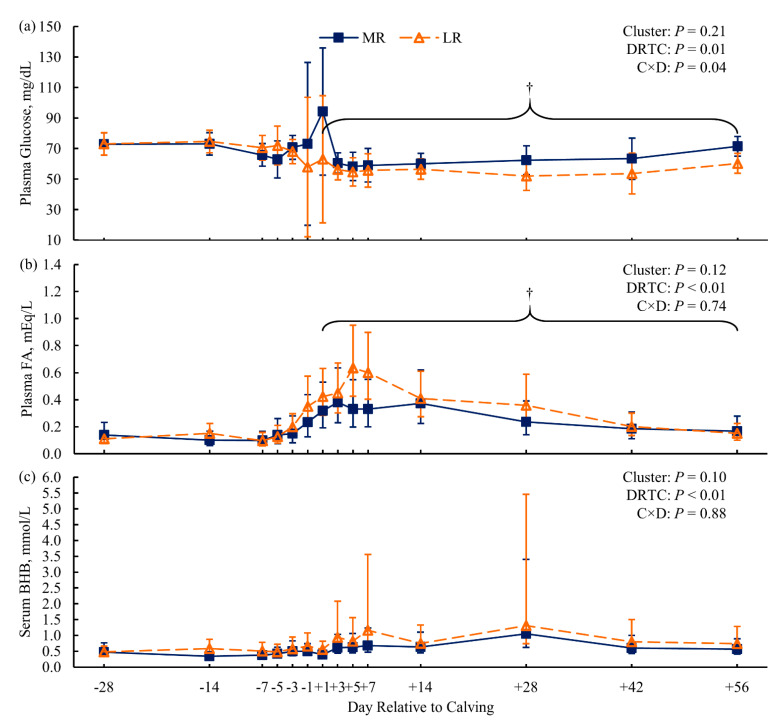
Blood fraction concentrations of glucose (panel **a**), fatty acids (FA; panel **b**), and β-hydroxybutyrate (BHB; panel **c**) for dairy cows more resistant (MR) or less (LR) resistant to lipid-related metabolic disorders (*n* = 3 cows per cluster). Data are presented as least squares means (points) and their 95% confidence intervals (error bars). Statistics for the fixed effects of cluster, day relative to calving (DRTC), and their interaction (C × D) are displayed in the top-right corner of each panel. Marginal evidence (†; *p* = 0.07, panel **a**; *p* = 0.06, panel **b**) for contrasts of cluster across postpartum samples (bracketed) are indicated within their respective panels.

For the resistance clusters within the KIP treatment, cows were individually feed-restricted after the +14 DRTC sampling until blood BHB ≥ 3.0 mmol/L. The number of days feed-restricted until achieving the threshold was 4, 8, and 13 d for the MR cows, and 0, 2, and 8 d for the LR cluster cows. A single LR cow was diagnosed with displaced abomasum and hypocalcemia by the herd veterinarian. Resistance clusters did not differ in BCS (*p* = 0.91; Figure 1d), but the MR cows had greater BW from +7 to +56 DRTC (*p* ≤ 0.07 for Bonferroni-adjusted simple effects; Figure 1b) compared to LR cows. Milk energy output and milk lactose yield were similar (*p* > 0.99 and *p* = 0.52, respectively; Supplementary Table S3) between resistance clusters. Marginal evidence of greater postpartum DMI (*p* = 0.09, contrast) for MR cows compared to LR cows was observed, contributing to the significantly (*p* = 0.01) attenuated negative energy balance observed for MR cows compared to LR cows (Figure 1f). Plasma glucose concentrations showed marginal evidence of being greater postpartum (*p* = 0.07, contrast) for MR cows than for LR cows (Figure 4a). Compared to their LR contemporaries, MR cows showed marginal evidence of lower concentrations of postpartum plasma FA (*p* = 0.06, contrast; Figure 4b) and serum BHB (*p* = 0.10; Figure 4c), as well as significant evidence of lower liver TG (*p* = 0.03; Figure 3b).

### 3.2. Differentially Expressed Genes and Enriched Metabolic Pathways

For the comparison of LS and MS clusters, the count of genes with FPKM > 0 was 13,151 genes at −28 DRTC, 13,011 genes at +1 DRTC, and 13,211 genes at +14 DRTC tested for differential expression (Table 1). Genes with significant (marginal) evidence on −28, +1, and +14 DRTC totaled 165 (58), 116 (31), and 199 (39), respectively (Supplementary Microsoft Excel File). A selection of genes with significant or marginal evidence for differential expression across all DRTC within the susceptibility cluster comparisons is listed in Table 2. Metabolic pathways enriched within the DEGs with significant (marginal) evidence numbered 0 (2), 4 (3), and 5 (2) for −28, +1, and +14 DRTC, respectively (Table 3).

Comparing the MR and LR cows, the count of genes with FPKM > 0 was 13,139 genes at −28 DRTC, 13,150 genes at +1 DRTC, and 12,718 genes at +14 DRTC tested for differential expression (Table 1). Significant (marginal) evidence for differential expression was found for 127 (30), 142 (50), and 102 (31) genes at −28, +1, and +14 DRTC, respectively (Supplementary Microsoft Excel File). A selection of genes with significant or marginal evidence for differential expression across all DRTC within the resistance cluster comparisons is listed in Table 4. Metabolic pathways enriched within the DEGs with significant (marginal) evidence numbered 0 (5), 6 (2), and 0 (0) for −28, +1, and +14 DRTC, respectively (Table 5).

## 4. Discussion

The purpose of this work is to provide novel insight into the genes and metabolic pathways integral to the pathology of LRMD. To that end, we retrospectively identified groups of periparturient dairy cows within a relatively normal dietary scenario and within a metabolically challenging dietary scenario, with apparently different metabolic statuses. These differences in metabolic status were determined via *k*-means clustering of cows based on postpartum liver and blood characterization as indicators of HYK and FLS [17,38,45]. It is important to acknowledge that the sample size for the cluster comparisons was relatively small (*n* = 3 cows/cluster), which can impede the detection of response differences or limit generalizability. Nevertheless, evidence for differences in lipid biomarkers—particularly liver TG and blood BHB—were found between clusters. Although nutrient partitioning is a normal physiological adaptation that may support feed efficiency, metabolic health, and productivity [46,47,48,49], dysregulation or imbalance can reflect metabolic disorders, which are the focus herein. Sequencing of the liver transcriptomes at several peripartum timepoints suggested numerous genes and metabolic pathways associated with LRMD at one or more timepoints. Therefore, this discussion will only concern the phenotypic characterization of the clusters within the original dietary treatment, along with a selection of genes, gene families, and metabolic pathways.

### 4.1. Phenotypic Characterization of Clusters

Cows within the CTL treatment were subject to typical pre- and postpartum diets for dairy cows, without additional imposed challenges [35]. Differential regulation of genes and metabolic pathways may underlie facets of liver metabolism that may predispose an individual cow to the progression of LRMD. The principal metabolic differences between the LS and MS cows were the greater serum BHB (*p* = 0.08; Figure 2c) and liver TG (*p* = 0.02; Figure 4a) concentrations observed for the MS cows. As mentioned, these metabolites serve as the primary biomarkers for HYK and FLS, with greater concentrations suggesting pathology [17,38,45]. Thus, the MS cluster appears to be in a less favorable metabolic condition and prone to LRMD. Greater plasma FA concentration is also a biomarker of LRMD, and would be expected in the MS cluster [39,50]. Although plasma FA were numerically greater for MS cows postpartum (Figure 2b), they was not significantly different from the LS cows. This lack of difference in FA is corroborated by the similar BW and BCS between the susceptibility clusters peripartum (Figure 1), suggesting unappreciable differences in the lipolysis of adipose TG between clusters. Additionally, these clusters did not differ in DMI, milk energy output, energy balance (Figure 1e), or plasma glucose (Figure 2a). Although these differences are in contrast to the present dogma of insufficient nutrient supply and over-mobilization of body energy reserves leading to LRMD [17,18,45], it is important to remember that the lack of differences here may be due to the small sample size with regard to metabolite analysis. Conversely, these data may suggest that some cows are susceptible to onset of LRMD despite the lack of energetic challenges that are classically associated with the dogma, and may be susceptible due to independent risk factors.

The KIP treatment imposed on the cows in the resistance clusters (MR and LR) was intended to predispose cows to LRMD. All cows in the KIP treatment progressed to a clinical HYK blood BHB threshold (BHB ≥ 3.0 mmol/L). Thus, the aim of metabolic clustering was to identify cows more resistant to the KIP treatment, with lower metabolite concentrations (plasma FA, serum BHB, and liver TG) or a greater number of feed restriction days until blood BHB ≥ 3.0 mmol/L. Consistent with this goal, dairy cows in the MR cluster had lower concentrations of serum BHB (*p* = 0.10; Figure 4c) and liver TG postpartum (*p* = 0.03; Figure 4b) compared to the LR cluster. Furthermore, MR cows required more feed restriction days to achieve BHB ≥ 3.0 mmol/L. Together, these data suggest that cows in the MR cluster were more resistant to LRMD than LR cows. Compared to LR cows, MR cows had greater postpartum BW (Figure 1b) and less negative energy balance postpartum (Figure 1f), suggesting that the MR cows did not mobilize as much of their endogenous energy reserves to support lactation nutrient requirements as the LR cows. Greater plasma FA concentration for the LR cows (Figure 4b) corroborates greater lipolysis of adipose tissue TG. The greater plasma glucose concentration for the MR cows postpartum (Figure 4a), with similar milk lactose output, may suggest that MR cows had greater hepatic gluconeogenesis or decreased peripheral glucose utilization compared to LR cows [1,8,51]. Overall, the MR cows appeared to experience a more favorable adaptation to lactation when a dietary challenge was imposed than the LR cows.

### 4.2. Inferred Differential Regulation of the Liver Transcriptome

Mobilization of adipose tissue TG as FA and their linear uptake by the liver results in a tremendous supply of liver FA for metabolism in dairy cows [52,53]. Oxidation of these FA in the hepatocyte mitochondria and peroxisomes to produce energy can also produce reactive oxygen species (ROS). Accumulation of hepatic ROS in early postpartum dairy cows can induce oxidative stress, characterized by excessive oxidation of proteins and lipids and induction of apoptosis [54,55]. Glutathione is a protein that can serve as an antioxidant, protecting cells from ROS-induced oxidative damage [26,55,56]. In the present experiment, the KEGG pathway glutathione metabolism was enriched in the DEGs identified in the comparison of susceptibility clusters on +1 DRTC (Table 3). The LS cows had greater expression of genes in the glutathione-centered antioxidant defense system than MS cows: *GSTA4* (2.6-fold), *GSTT1*(2.0-fold), and *GSTA1* (2.5-fold); these genes catalyze glutathione conjugations with electrophilic compounds (i.e., drugs, xenobiotics, lipid hydroperoxides), and some have glutathione peroxidase activity [57,58,59]. Thus, the LS cows appear to have improved their antioxidation capacity compared to their MS contemporaries.

Eicosanoids, a subcategory of oxylipids, are signaling molecules made by the enzymatic or non-enzymatic oxidation of arachidonic acid or other polyunsaturated FA [54,60]. These molecules can have either pro- or anti-inflammatory properties, while influencing oxidative stress [54]. Eicosanoids can directly promote oxidative stress through the production of ROS, or indirectly via the production of ROS during biosynthesis. Some eicosanoids—such as 15-deoxy-delta 12 and 14-Prostaglandin J2—exert antioxidant effects by directly or indirectly targeting and decreasing ROS production by other cellular metabolic processes (i.e., mitochondrial oxidation) [54,60]. At +1 DRTC, the KEGG pathways linoleic acid metabolism, alpha-linoleic acid metabolism, and arachidonic acid metabolism were enriched for the comparison of the LRMD resistance cluster (Table 5). All of the specific DEGs were expressed more in the livers of LR cows: *PLB1* (36.8-fold), *PLA2* (infinity (∞)-fold), *PLA2G2A* (∞-fold), *CYP2B6* (2.6-fold), and *CYP2J2* (4.3-fold). The inferred promotion of eicosanoid synthesis may be a byproduct of the greater ROS production and pathology of LRMD. However, the greater eicosanoid production may be an adaptation by the LR cows to accelerate the termination of the liver’s inflammatory state.

The role of the immune system in the progression of metabolic syndromes is an emerging scientific field in dairy science [61,62]. In humans, there appears to be crosstalk between metabolism and the immune response, which furthers the progress of non-alcoholic fatty liver to steatohepatitis and cirrhosis [63]. In the present experiment, many of the most consistent DEGs across the DRTC were the immunity-related genes involved in expressing components of the major histocompatibility complex (MHC; classes I and II) and genes in the interferon-inducible protein (IFI) family for LRMD susceptibility and resistance comparisons.

The MHC molecules are responsible for the presentation of antigens on the cell surface, and recruit other mechanisms of the innate immune response when antigens represent “non-self” proteins [64]. Expression of MHC molecules—particularly MHC class II—has been implicated in the pathology of liver metabolic and inflammatory disease in humans [63,64]. In our data, the expression of MHC elements was found in the cell adhesion molecules pathway at −28 and +1 DRTC for the susceptibility clusters and −28 DRTC for the resistance cluster comparison. Of note, *bovine lymphocyte antigen-DQB* (*BOLA-DQB*)—an MHC class II protein—was one of the few DEGs observed at every DRTC for the LRMD susceptibility and resistance cluster comparisons. Liver tissue is composed of several cell types, including hepatocytes, hepatic stellate cells, Kupffer cells, and sinusoidal cells [65]. Expression of MHC class II molecules is typically restricted to professional antigen-presenting cell types such as Kupffer cells, and not hepatocytes [66]; however, hepatocytes from humans with clinical hepatitis do express MHC class II molecules [67], which may be capable of attracting CD4+ T lymphocytes [64]. It is theorized that ROS-induced peroxidation of cellular components, such as proteins and lipids, can induce MHC expression and presentation of the oxidized component antigens to the cell surface [63]. The subsequent recruitment of CD4+ T lymphocytes and cytokine expression promote the apoptosis and clearance of ROS-damaged hepatocytes by the immune system [63,64]. Even though hepatocytes are the predominant liver cell type, differential expression of these genes may be found within Kupffer cells. Fatty acid binding to Kupffer Toll-like receptors has been demonstrated to promote macrophage recruitment through the C-JNK and NF-κB pathways, promoting liver lipid synthesis and mitochondrial dysfunction [68,69]. Interestingly, *BOLA-DQB* and other MHC components generally had greater expression for the LS and LR cows than for the MS and MR cows, respectively. Considering that the LS group was the metabolically preferable group within the susceptibility cluster, we did not expect the relative directionality of these DEGs to be similar to LR—the less preferable resistance cluster. It may be the case that cows not experiencing additional dietary challenges benefit from sensitive clearance of ROS-damaged hepatocytes, while cows challenged with additional prepartum energy may suffer from an excessive quantity of ROS-damaged cells or oversensitive presentation of antigens, resulting in disproportional apoptosis and clearance, but this would require further examination.

Interferons are cytokines that are best known for their secretion in response to viral infections, but have been implicated in the pathology of non-alcoholic fatty liver disease in humans [70,71]. The IFI and other interferon-stimulated genes are downstream effectors of interferon [72]. Similar to the MHC genes, IFI genes—specifically *IFI6*, *IFI27*, *IFI44*, and *IFI44L*—had greater relative expression (range of 2.0- to 5.6-fold) for LS and LR cows than for MS and MR cows, respectively, across all DRTC. It is possible that the IFI genes serve as the upstream regulators promoting the expression of the MHC molecules [72], and promote the clearance of ROS-damaged hepatocytes, as previously discussed.

Serum amyloid A (SAA) is an acute phase response protein associated with the inflammatory cascade that has been previously associated with HYK [73,74,75]. The isoforms SAA1 and SAA2 are generally considered to be the predominant proinflammatory proteins expressed in the liver, while SAA3 has generally been viewed as an adipokine [73,75]. Expression of *SAA3* was significantly greater for LS than MS cows at −28 DRTC (6.1-fold), but LS cows had significantly lower *SAA3* expression (3.2-fold) at +1 DRTC than MS cows. At +14 DRTC, the LS cluster had greater expression of *SAA2* (6.1-fold), *SAA4* (1.7-fold), and *LOC104968478* (21.1-fold) than the MS cluster. The LR cluster had consistently greater expression of all SAA isoforms than the MR cluster, ranging from 4- to 9.8-fold. Comparison of resistance clusters is consistent with previous works associating greater abundance of SAA and states with LRMD [74,76]. However, the susceptibility cluster comparison suggests a more nuanced regulation of SAA in the absence of additional dietary challenges. It has been previously suggested that a priming of the inflammatory response may exert protective effects in peripartum dairy cows [77], which the relative expression pattern of SAA3 for the susceptibility clusters may support.

### 4.3. Insight into LRMD Pathology

There is considerable variation in the metabolic health of dairy cows peripartum; this is evident in the variable prevalence of LRMD and their comorbidities [12,38,78]. In addition, the variation in the occurrence and severity of LRMD is still evident when cows are subjected to a dietary challenge [32,35]. Our retrospective clustering of dairy cows based on lipid metabolites within the CTL and KIP dietary treatments empirically identified groups of cows with divergent metabolic health, as previously discussed. The apparent presence of these divergent groups within both dietary conditions indicated two potential control points for LRMD pathology: susceptibility to LRMD incidence, and resistance to LRMD severity. Even though the gross pathology of LRMD pertaining to lipid metabolism and gluconeogenesis has been investigated, there has been limited investigation into the divergent metabolic regulation responsible for these LRMD susceptibility or resistance control points. The evaluation of the liver transcriptome through RNA sequencing allowed for a more holistic evaluation of what unique and shared metabolic pathways underpin these control points [79].

**Figure 5:**
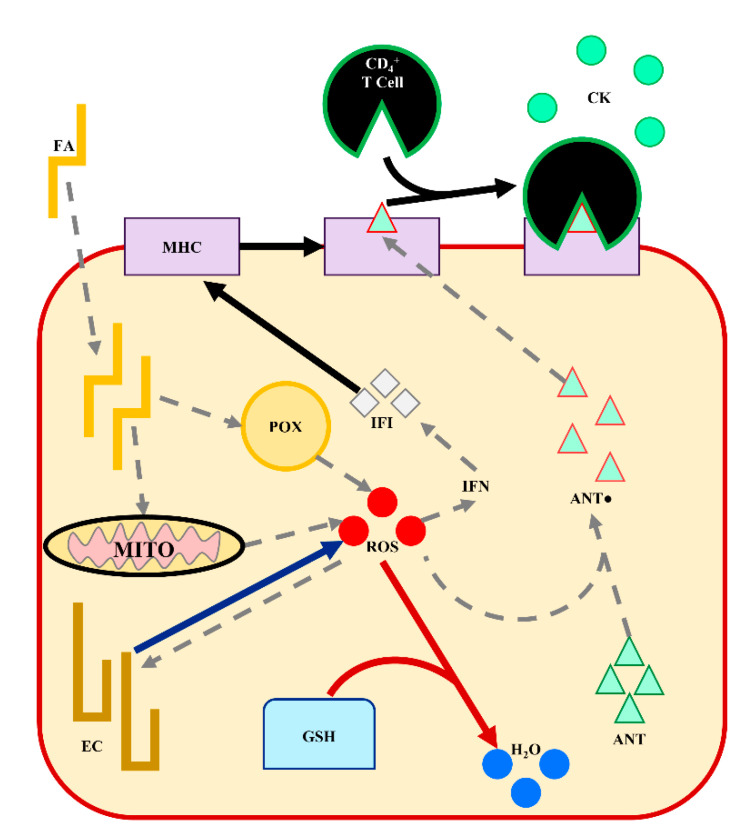
A working model of differentially regulated metabolic pathways in the liver of dairy cows that contribute to the pathology of lipid-related metabolic disorders. Fatty acids (FA) entering the hepatocyte are oxidized in the mitochondria (MITO) and peroxisomes (POX), producing energy and reactive oxygen species (ROS). Interferon (IFN) production is promoted by ROS, stimulating major histocompatibility complex (MHC) expression. Antigens (ANT) are oxidized (ANT●) by ROS. The ANT● are presented by the MHC promoting CD4+ T lymphocyte recruitment and cytokine (CK) production. Eicosanoids (EC) are formed by FA oxidation by ROS, and may promote ROS production. Glutathione (GSH) reduces ROS and other oxidized products. Arrows demonstrate the directionality and specificity of metabolic pathways: pathways not differentially regulated are grey dashed lines (----), pathways upregulated in cows less resistant to LRMD are blue (⸺⸺), pathways upregulated in cows less susceptible to LRMD are red (⸺⸺), and pathways generally upregulated in cows less susceptible and less resistant are solid black (⸺⸺).

The genes and metabolic pathways presented in this work appear to be centered around hepatic adaptation to the lipotoxic effects of ROS through the innate immune system and inflammatory response. As discussed, the intersection of nutrient metabolism, immunity, and inflammation in the peripartum physiological adaptations and LRMD pathology has been a growing cross-disciplinary topic in dairy science [62,76]. These observations support further investigation into immunometabolism—specifically on the contributions of glutathione metabolism, eicosanoid metabolism, and MHC molecules in liver tissue. The observed regulation for these pathways is summarized in Figure 5. Interestingly, the susceptibility and resistance comparisons each had a unique inferred metabolic pathway at +1 DRTC. Glutathione metabolism was unique to the susceptibility comparison, while eicosanoid metabolism was a unique to the resistance comparison (Table 3; Table 5). Of course, these differences could be due to differences in the expressed genes available for testing between these +1 DRTC comparisons (Table 1). The relevance of MHC molecules and the innate immune response is apparently shared across the susceptibility and resistance comparisons. However, the relative expression of the DEGs and EMPs (i.e., BOLA–DQB) was greater for the metabolically preferable susceptibility cluster (LS) and the metabolically less preferable resistance cluster (LR). This discordant direction of expression across LRMD susceptibility and resistance comparisons suggests that experimental conditions—especially induction protocols—require nuanced interpretation. While some differentially regulated genes and pathways may be shared between “natural” LRMD development and induction, the observed response directionality may reflect adaptive mechanisms to mitigate the severity of LRMD rather than their progression or risk. Additionally, there may be unique mechanisms to the natural pathology of LRMD, as well as mechanisms that resist the progression of LRMD.

## 5. Conclusions

There is substantial individual variation in the metabolic responses of dairy cows to peripartum conditions, suggesting that the underpinning regulation of key metabolic pathways may confer susceptibility or resistance to LRMD. We empirically grouped multiparous Holstein cows within normal and challenged peripartum dietary conditions based on their blood lipid metabolite profiles and liver TG contents. This approach revealed two metabolic health groups within each dietary condition, suggesting differential susceptibility to LRMD incidence or resistance to LRMD induced by a dietary challenge. These metabolic differences were realized in the liver transcriptomes of these cow groups, with the inferred differential metabolism highlighting the role of the inflammatory response and innate immunity in LRMD pathology. Novel insights included the differential regulation of MHC molecules and IFI, which may aid the response to the ROS-induced cellular damage that occurs in liver tissue peripartum. Furthermore, the contributions of liver glutathione and eicosanoid metabolism to LRMD pathology immediately postpartum appear to be of greater biological importance relative to dietary condition—normal and challenged, respectively. Future work should build on the contributions of these specific mechanisms in liver cell types and delineate their dependence on specific dietary conditions.

## Figures and Tables

**Table 1:** The numbers of genes tested within days relative to calving (DRTC; diagonal) that were shared within and across comparison of clusters ^1^.

		Susceptibility			Resistance		
	DRTC	−28	+1	+14	−28	+1	+14
Susceptibility	−28	13,151	12,752	12,837	12,833	12,757	12,519
	+1	21,009	13,011	12,784	12,670	12,744	12,512
	+14	20,891	20,978	13,211	12,768	12,774	12,620
Resistance	−28	20,962	20,939	20,834	13,139	12,736	12,518
	+1	20,874	21,001	20,828	20,865	13,150	12,525
	+14	21,065	21,198	21,103	21,076	21,071	12,718

^1^ The number of tested genes shared (pairwise) across DRTC and across cluster comparisons (susceptibility or resistance) are above the diagonal, while the number of untested genes shared are below the diagonal. There were 34,411 genes that were potentially tested across the *Bos taurus* genome (release 106, ARS-UCD 1.2).

**Table 2:** Fold change ^1^ of genes with significant or marginal evidence of differential expression at all days relative to calving in liver samples from cows less or more susceptible to lipid-related metabolic disorders ^2^.

Gene	Symbol	−28	+1	+14
BOLA class I histocompatibility antigen, alpha chain BL3-6	*BOLA*	−1.3	−1.3	−1.4
Major histocompatibility complex, class II, DQ beta	*BOLA-DQB*	−2.2	−3.0	−3.0
Coiled-coil domain-containing 80	*CCDC80*	−5.0	−1.4	−3.4
C-C motif chemokine 14 precursor	*CCL14*	−1.7	−1.9	−1.6
B-cell receptor CD22	*CD22*	1.4	1.1	0.9
Acetylcholine receptor subunit epsilon	*CHRNE*	2.3	1.3 ^†^	1.5
C-type lectin domain family 4 member F	*CLEC4F*	−1.3	−1.3 ^†^	−1.5
GTPase IMAP family member 6	*GIMAP6*	−1.3	−1.7	−1.7
GTPase IMAP family member 8	*GIMAP8*	−2.2	−2.0	−2.4
ISG15 ubiquitin-like modifier	*ISG15*	−1.6	−1.0 ^†^	1.1
Methylenetetrahydrofolate dehydrogenase 1-like	*MTHFD1L*	−1.4	−1.3 ^†^	−1.6
Olfactory receptor 4 × 2	*OR4S1*	1.0	0.9 ^†^	0.8
Prodynorphin	*PDYN*	−2.4	−2.9	−2.2
Secreted frizzled-related protein 2	*SFRP2*	5.6	4.4	2.0
Pulmonary surfactant-associated protein A precursor	*SFTPA1*	−3.7	−2.1	−1.6
Teneurin transmembrane protein 1	*TENM1*	−2.1	−1.3 ^†^	−0.9

^1^ Values represent the log_2_-transformed fold change within each timepoint. Positive values indicate greater expression for more susceptible than for less susceptible cows, and vice versa for negative values. ^2^ Genes were considered to have significant evidence of differential expression within days relative to calving (−28, +1, and +14) when *Q* ≤ 0.05 (*p*-value corrected for multiplicity by false discovery rate); meanwhile, marginal evidence (^†^) was declared at 0.05 < *Q* ≤ 0.10.

**Table 3:** Kyoto Encyclopedia of Genes and Genomes metabolic pathways enriched within the genes with significant or marginal evidence of differential expression in liver samples from cows less or more susceptible to lipid-related metabolic disorders ^1^.

DRTC	ID	Metabolic Pathway	FE	*Q*-Value	Gene Symbols ^2^
−28	bta04514	Cell adhesion molecules (CAMs)	5	0.098	*MGC126945*, *ITGAV*, *NRCAM*, *LOC534578*, *CD22*, *BOLA-DQB*, *LOC100848815*
	bta04145	Phagosome	4	0.098	*SEC61G*, *SFTPA1*, *MRC2*, *MGC126945*, *ITGAV*, *SEC61B*, *BOLA-DQB*
	bta03320	PPAR signaling pathway	6	0.136	*SCD*, *CYP8B1*, *APOA5*, *SLC27A6*, *FADS2*
+1	bta05204	Chemical carcinogenesis	7	0.023	*LOC615303*, *CYP1A1*, *GSTA4*, *GSTT1*, *GSTA1*, *CYP2C87*
	bta00480	Glutathione metabolism	8	0.026	*ODC1*, *GSTA4*, *GSTT1*, *ANPEP*, *GSTA1*
	bta00980	Metabolism of xenobiotics by cytochrome P450	7	0.026	*LOC615303*, *CYP1A1*, *GSTA4*, *GSTT1*, *GSTA1*
	bta05164	Influenza A	4	0.04	*RSAD2*, *OAS1X*, *DDX39B*, *DDX58*, *LOC784541*, *BOLA-DQB*, *LOC100848815*
	bta04141	Protein processing in endoplasmic reticulum	3	0.079	*CKAP4*, *HSPA5*, *HYOU1*, *DNAJB11*, *MOGS*, *SEC23B*, *WFS1*
	bta00982	Drug metabolism-cytochrome P450	6	0.079	*LOC615303*, *GSTA4*, *GSTT1*, *GSTA1*
	bta04514	Cell adhesion molecules (CAMs)	4.0	0.085	*NRCAM*, *LOC534578*, *CD22*, *BOLA-DQB*, *LOC100848815*
+14	bta04512	ECM-receptor interaction	9	6.3 × 10^−5^	*COL6A3*, *COL6A1*, *COL5A1*, *COL5A3*, *COL3A1*, *COL1A1*, *COL1A2*, *COL5A2*, *VWF*, *TNXB*
	bta04974	Protein digestion and absorption	10	6.8 × 10^−5^	*COL6A3*, *COL6A1*, *COL5A1*, *COL5A3*, *COL3A1*, *COL1A1*, *COL1A2*, *COL5A2*, *COL15A1*
	bta04611	Platelet activation	4.0	0.046	*COL5A1*, *COL5A3*, *ADCY1*, *COL3A1*, *COL1A1*, *COL1A2*, *COL5A2*, *VWF*
	bta04510	Focal adhesion	3	0.046	*COL6A3*, *COL6A1*, *COL5A1*, *COL5A3*, *COL3A1*, *COL1A1*, *COL1A2*, *COL5A2*, *VWF*, *TNXB*
	bta05146	Amoebiasis	5	0.046	*COL5A1*, *COL5A3*, *ADCY1*, *COL3A1*, *COL1A1*, *COL1A2*, *COL5A2*
	bta04923	Regulation of lipolysis in adipocytes	7	0.058	*PTGER3*, *ADCY1*, *FABP4*, *LIPE*, *IRS2*
	bta04151	PI3K–Akt signaling pathway	3	0.058	*IFNAR1*, *COL6A3*, *COL6A1*, *COL5A1*, *COL5A3*, *LPAR1*, *COL3A1*, *COL1A1*, *COL1A2*, *COL5A2*, *VWF*, *TNXB*

^1^ Enrichment analysis was performed within days relative to calving (DRTC) using the Database for Annotation, Visualization, and Integrated Discovery (version 6.8) by comparing a list of genes with significant or marginal evidence (*Q* ≤ 0.10; *p*-value adjusted for multiplicity by the false discovery rate method) to a custom background list including all tested genes within a DRTC. Fold enrichment (FE) and Fisher’s exact statistics were extracted; tests were corrected for multiplicity via the false discovery rate method (*Q*-value). ^2^ Annotation of gene transcripts and affiliated gene symbols are based on the *Bos taurus* reference genome (release 106, ARS-UCD 1.2).

**Table 4:** Fold change ^1^ of genes with significant or marginal evidence of differential expression at all days relative to calving in liver samples from cows more or less resistant to lipid-related metabolic disorders ^2^.

Gene	Symbol	−28	+1	+14
Major histocompatibility complex, class II, DQ beta	*BOLA-DQB*	1.9	2.1	2.6
GTPase IMAP family member 4	*GIMAP4*	−0.8 ^†^	−1.2	−1.2
17-Beta-hydroxysteroid dehydrogenase 13	*HSD17B13*	1.2	1.8	1.2
Interferon-induced protein 44	*IFI44*	1.3	1.8	1.3
Interferon alpha-inducible protein 6	*IFI6*	1.8	2.6	1.0
MHC Class I JSP.1	*JSP.1*	0.8 ^†^	1.1	0.7 ^†^
Myomesin 1	*MYOM1*	0.9	1.8	0.8
2′,5′-Oligoadenylate synthetase 1, 40/46kDa	*OAS1X*	2.3	1.8	1.4
Proto-oncogene tyrosine-protein kinase ROS	*ROS1*	−1.1	−1.4	−3.0

^1^ Values represent the log_2_-transformed fold change within each timepoint. Positive values indicate greater expression for less resistant than for more resistant cows, and vice versa for negative values. ^2^ Genes were considered to have significant evidence for differential expression within days relative to calving (−28, +1, and +14) when *Q* ≤ 0.05 (*p*-value corrected for multiplicity by false discovery rate); meanwhile, marginal evidence (^†^) was declared at 0.05 < *Q* ≤ 0.10.

**Table 5:** Kyoto Encyclopedia of Genes and Genomes metabolic pathways enriched within the genes with significant or marginal evidence of differential expression in liver samples from cows more or less resistant to lipid-related metabolic disorders ^1^.

DRTC	Metabolic Pathway	FE	*Q*-Value	Gene Symbols ^2^
−28	Cytokine–cytokine receptor interaction	4.6	0.099	*PF4*, *CXCR2*, *CXCR1*, *IL1R2*, *CCR1*, *CSF2RB*
	Cell adhesion molecules (CAMs)	4.5	0.099	*VCAN*, *JSP.1*, *SELL*, *LOC534578*, *BOLA-DQB*
	Viral myocarditis	6.4	0.099	*JSP.1*, *MYH7*, *CD55*, *BOLA-DQB*
	Chemokine signaling pathway	3.5	0.099	*PF4*, *NCF1*, *CXCR2*, *CXCR1*, *CCR1*, *CCL24*
	Hematopoietic cell lineage	5.7	0.099	*MS4A1*, *LOC515418*, *IL1R2*, *CD55*
+1	Metabolic pathways	14.1	0.041	*IDI1*, *HAL*, *SDS*, *LOC511161*, *KYAT1*, *ALPI*, *FOLH1B*, *LOC615045*, *BCO1*, *OAT*, *GCSH*, *ASAH2*, *PLB1*, *FDPS*, *FUT1*, *HDC*, *AKR1B10*, *MBOAT2*, *ACSS2*, *MTHFD1L*, *ADH4*, *ENPP3*, *B4GALT4*, *PLA2G2A*, *CYP2J2*, *LOC100125266*, *CYP2B6*
	Arachidonic acid metabolism	2.6	0.041	*PLB1*, *LOC615045*, *PLA2G2A*, *CYP2B6*, *CYP2J2*
	Linoleic acid metabolism	2.1	0.041	*PLB1*, *LOC615045*, *PLA2G2A*, *CYP2J2*
	Autoimmune thyroid disease	2.1	0.041	*JSP.1*, *LOC524810*, *LOC100300716*, *BOLA-DQB*
	Allograft rejection	2.1	0.041	*JSP.1*, *LOC524810*, *LOC100300716*, *BOLA-DQB*
	Viral myocarditis	2.6	0.041	*JSP.1*, *LOC524810*, *LOC100300716*, *CD55*, *BOLA-DQB*
	Intestinal immune network for IgA production	2.1	0.083	*LOC524810*, *PIGR*, *LOC100300716*, *BOLA-DQB*
	alpha-Linolenic acid metabolism	1.6	0.086	*PLB1*, *LOC615045*, *PLA2G2A*

^1^ Enrichment analysis was performed within days relative to calving (DRTC) using the Database for Annotation, Visualization, and Integrated Discovery (version 6.8) by comparing a list of genes with significant or marginal evidence (*Q* ≤ 0.10; *p*-value adjusted for multiplicity by the false discovery rate method) to a custom background list including all tested genes within a DRTC. Fold enrichment (FE) and Fisher’s exact statistics were extracted; tests were corrected for multiplicity by the false discovery rate method (*Q*-value).^2^ Annotation of gene transcripts and affiliated gene symbols are based on the *Bos taurus* reference genome (release 106, ARS-UCD 1.2).

## Data Availability

Data are available upon request from the corresponding author.

## Supplementary Materials

The following are available online at https://www.mdpi.com/article/10.3390/ani11092558/s1. Supplementary Table S1. Ingredient and nutrient composition of pre- and postpartum experimental diets., Supplementary Table S2. Least squares means (LSM) and 95% confidence intervals (CI) of phenotypic responses for cow less (LS) or more susceptible (MS) to lipid-related metabolic disorders., Supplementary Table S3. Least squares means (LSM) and 95% confidence intervals (CI) of phenotypic responses for cows more (MR) or less resistant (LR) to lipid-related metabolic disorders.
