# Supplementary material for: Novel Facets of the Liver Transcriptome Are Associated with the Susceptibility and Resistance to Lipid-Related Metabolic Disorders in Periparturient Holstein Cows
Source: Animals (Basel). 2021 Aug 31;11(9):2558. doi: 10.3390/ani11092558 (PMC8470208; doi:10.3390/ani11092558)
Supplement: Supplementary file 1 [file animals-11-02558-s001.zip › animals-1320038-supplementary_AUproof.pdf]

# Novel facets of the liver transcriptome are associated with the susceptibility and resistance to lipid-related metabolic disorders in periparturient Holstein cows

**Ryan S. Pralle [1,2], Wenli Li [3,*], Brianna N. Murphy [3], Henry T. Holdorf [1], and Heather M. White [1,*]**

[1]Department of Animal and Dairy Sciences, University of Wisconsin-Madison, Madison, WI 53706, USA; praller@uwplatt.edu (R.S.P.); hholdorf@wisc.edu (H.T.H.); heather.white@wisc.edu (H.M.W.)
[2]Current Affiliation: School of Agriculture, University of Wisconsin-Platteville, Platteville, WI 53818, USA; praller@uwplatt.edu
[3]USDA-Agricultural Research Service, Dairy Forage Research Center, Madison, WI 53706, USA; wenli.li@usda.gov (W.L.); bmurphy8@wisc.edu (B.N.M.)
[*]Co-Corresponding Authors: wenli.li@usda.gov; Tel.: +1-608-890-0056 (W.L.); heather.white@wisc.edu; Tel.: +1-608-263-7783 (H.M.W.)

**Supplementary Table S1.** Ingredient and nutrient composition of pre- and postpartum experimental diets.

| Diet Component | Prepartum[1] | Postpartum[2] |
|---|---|---|
| Ingredient, % DM | | |
| Straw | 29.63 | – |
| Corn silage | 51.12 | 28.36 |
| Concentrate Mix[3] | 19.25 | 36.07 |
| Alfalfa silage | – | 29.78 |
| Cottonseed | – | 5.80 |
| Chemical composition | | |
| DM, % | 43.70 | 48.71 |
| CP, % DM | 12.65 | 17.00 |
| NDF, % DM | 43.55 | 29.90 |
| Lignin, % DM | 4.62 | 4.18 |
| EE, % DM | 2.63 | 5.05 |
| NFC, % DM | 34.36 | 42.69 |
| Ash, % DM | 7.34 | 7.36 |
| $NE_L$, Mcal/kg DM | 1.42 | 1.65 |

[1]Cows assigned to the ketosis induction protocol received 6 kg of dry cracked corn (90.2 % DM, 1.82 Mcal/kg DM) as a top-dress from -28 expected days relative to calving to parturition.

[2]Control and ketosis induction treatments received the same postpartum diet; however, ketosis induction cows were feed restricted to 80% of *ad libitum* intake from +14 days relative to calving until their blood β-hydroxybutyrate concentration was ≥ 3.0 mmol/L.

[3]Prepartum concentrate mix: Soybean meal 46 % CP (86.3 %), CaCO3 (3.37 %), Ca(H2PO4)2 (1.65 %), and a premix (8.68 %) composed of: CaSO4 (23.20 %), NaCl (13.85 %), CaCO3 (12.00 %), MgO (11.85 %), MgSO4 (11.80 %), CaHPO4 (21.0 %), mineral oil (1.0 %), selenium yeast 3000 (0.56 %, Prince Agri Products, Teaneck, NJ), Rumensin-90 (0.43%, Elanco Animal Health, Greenfield, IN), biotin (0.42%, DSM Nutritional Products, Belvidere, NJ), vitamin A (439.1 KIU/kg), vitamin D3 (132.7 KIU/kg), and vitamin E (6.4 KIU/kg). Postpartum concentrate mix: contained fine ground corn (55.78 %), canola meal (14.75 %), distillers grain (8.88 %), soy hull pellet (4.38 %), exceller meal 8.88 %, Quality Roasting Inc., Valders, WI), CaCO3 (2.25 %), NaHCO3 (2.25 %), grease (0.88 %), Urea (0.53 %), MgO (0.40 %), and a premix (1.05 %) composed of: Cl (51.2 %), Na (34 %), Ca (0.5 %), S (0.09 %), Co (78.2 ppm), Cu (4,871 ppm), I (469 ppm), Mn (14,382 ppm), Se (89.5 ppm), Zn (20,708 ppm), vitamin A (2055.7 KIU/kg), vitamin D3 (411.1 KIU/kg), vitamin E (8.7 KUI/kg), Rumensin-90 (1.8%, Elanco Animal Health, Greenfield, IN), and biotin (0.8%, DSM Nutritional Products, Belvidere, NJ).

## SUPPLEMENTAL METHODS

Daily feed offered and refused was recorded by trained herd staff for each individual cow and daily feed intakes were determined by calculation. Individual feed ingredients and total mixed ration samples were collected weekly. Feed ingredients were dried in a 55° C forced-air oven for 48 h, ground through a 1 mm screen by a Wiley Mill (Thomas Scientific, Swedesboro, NJ), and equal mass of dry, ground feed were composited by month. Composited samples were analyzed for composition by a commercial laboratory (Dairyland Labs, Arcadia, WI). Daily intake of net energy of lactation ($NE_L$) was calculated based on published equations employed in the NRC (2001) dairy model software using the chemical composition of composited feed ingredients. The maintenance $NE_L$ requirement was calculated as described by the NRC[50]: $0.08 \times BW^{0.75}$. Milk $NE_L$ yield was calculated based on the following NRC equation: [(0.0929 × % milk fat) + (0.0563 × % milk true protein/0.93) + (0.0395 × % milk lactose)] × milk yield. Pregnancy $NE_L$ requirement was calculated based on the following NRC equation[50]: [(0.00318 × day of gestation – 0.0352) × (calf BW / 45)] / 0.218. Net energy balance was calculated using the weekly averages of $NE_L$ requirements and intake with the following equation: $NE_L$ intake – milk $NE_L$ yield – maintenance $NE_L$ – pregnancy $NE_L$.

Milking occurred 2x daily and milk yield was recorded at every milking (BouMatic Xcalibur Herringbone, BouMatic, Madison, WI). For composition analysis, composite milk samples were collected at 4 consecutive milkings each week, preserved with 2-bromo-2-nitropropane-1,3-diol, and refrigerated at 4° C until shipping (within 1 d of last consecutive sample) for analysis. Milk composition of fat, protein, lactose, solids not fat, milk urea nitrogen, and SCC were determined by a DHIA laboratory (AgSource, Menominee, WI). All milk samples were preheated to 40° C and mixed before analysis of milk fat and milk protein by FTIR using the Foss MilkoScan FT+ (Foss Analytical, Hillerød, Denmark) in accordance with the instrument manufacturer's instructions and ISO 9622/ IDF 141:2013 (AOAC official method 972.16; AOAC International, 2016). Analysis of SCC was performed using Fossomatic FC (Foss Analytical). Per the DHIA's standard operating procedures, milk samples were analyzed on equipment that is calibrated weekly with 12 standards, and standards are rechecked daily and hourly with a subset of 6 of the 12 standards. Intra-assay coefficients of variation for all variables were maintained at < 7%. Inter-assay coefficients of variation are not available for all variables; however, inter-assay coefficients of variation for fat and protein are maintained at < 2% and < 1.5%, respectively.

Body weights, body condition score (BCS), blood samples, and liver biopsies were collected at -28, -14, +1, +14, +28, +42, and +56 days relative to calving (DRTC) and additional blood samples were taken at -7, -5, -3, -1, +3, +5, +7 DRTC. Two trained individuals independently recorded BCS using a five-point scale and the scores were

averaged within an observation. Blood samples were collected by venipuncture of the coccygeal vessels before feeding at approximately 0800 hours into evacuated tubes with or without additive.

Serum was separated from blood collected in tubes without additive (BD Vacutainer, Franklin Lakes, NJ) after centrifuging at 2,500 x *g* for 15 minutes at room temperature. Plasma was separated from blood collected in an evacuated tube containing potassium oxalate and 4% sodium fluoride (BD Vacutainer, Franklin Lakes, NJ) by centrifuging at 2,000 x *g* for 15 minutes at 4° C. Serum and plasma aliquots were stored at -20° C until metabolite analysis. Plasma glucose and serum β-hydroxybutyrate (BHB) concentrations were quantified in their respective aliquots using Catachem VETSPEC reagents on the Catachem Well-T AutoAnalyzer (Catachem, Awareness Technologies, Oxford, CT). All standards were within expected, calibrated ranges provided by the manufacturer during the calibration event (Catachem, Oxford, CT). Samples were read by the autoanalyzer in cuvettes in duplicate. Methods for glucose (C124-06, Catachem), BHB (C444-0A, Catachem), and TG (C116-0A, Catachem), respectively. Plasma fatty acid (FA) concentration was quantified enzymatically using a plate adaptation of the Catachem assay (C514-0A). Aliquots of a reference pool sample, respective to each sample type, were utilized for assay quality control. Intra-assay coefficient of variation never exceeded 10% for the quantification of the preceding blood fraction and liver metabolites. Inter-assay coefficients of variation were 5.9%, 6.5%, and 5.6%, for plasma glucose, plasma FA, and serum BHB, respectively.

Liver samples (~750 mg) were obtained by blind percutaneous biopsy utilizing a custom-built trocar. Biopsy samples were immediately rinsed with saline, aliquoted into tubes (~250 mg liver per tube), frozen in liquid nitrogen, and stored at -80° C until further analysis of liver TG. As described by Caputo Oliveira et al. (2020), liver TG content was quantified by colorimetric assay of Folch-extracted product and expressed as a % of dry matter.

**Supplementary Table S2.** Least squares means (LSM) and 95% confidence intervals (CI) of phenotypic responses for cow less (LS) or more susceptible (MS) to lipid-related metabolic disorders.[1]

| | LS | | MS | | *P*-value | | |
|---|---|---|---|---|---|---|---|
| **Response**[2] | **LSM** | **95% CI** | **LSM** | **95% CI** | **Cluster** | **Time** | **Cluster×Time** |
| BW, kg | 748.20 | [717.07, 779.31] | 745.30 | [714.11, 776.47] | 0.85 | <0.01 | 0.17 |
| BCS, pts | 3.29 | [3.66, 3.02] | 3.28 | [3.64, 3.01] | 0.93 | <0.01 | 0.14 |
| DMI, kg/d | 22.12 | [19.56, 24.36] | 23.24 | [20.84, 25.35] | 0.41 | <0.01 | 0.89 |
| Milk Energy, Mcal/d | 40.78 | [34.25, 46.21] | 38.98 | [31.99, 44.68] | 0.59 | 0.09 | 0.97 |
| EBAL, Mcal | | | | | | | |
| Prepartum | 9.08 | [5.21, 12.95] | 8.79 | [4.92, 12.66] | 0.89 | <0.01 | 0.74 |
| Postpartum | -11.89 | [-16.95, 6.83] | -8.54 | [-13.6, 3.49] | 0.28 | 0.03 | 0.63 |
| Plasma glucose, mg/dL | 65.18 | [61.03, 69.34] | 64.05 | [60.03, 68.08] | 0.62 | <0.01 | 0.70 |
| Plasma FA, mEq/L | 0.23 | [0.14, 0.37] | 0.29 | [0.18, 0.46] | 0.39 | <0.01 | 0.38 |
| Serum BHB, mmol/L | 0.55 | [0.49, 0.60] | 0.62 | [0.56, 0.69] | 0.08 | 0.08 | 0.69 |
| Liver TG %, DM | 4.27 | [2.81, 6.47] | 8.91 | [5.79, 13.72] | 0.02 | <0.01 | 0.38 |

[1]Cow disposition to lipid-related metabolic disorders was determined based on K-means clustering of cows originally assigned to a control dietary treatment based on lipid metabolite concentrations in blood fractions (plasma or serum) and liver tissue (n = 3 cows per cluster).

[2]BW = body weight, BCS = body condition score, DMI = dry matter intake, EBAL = calculated energy balance, FA = fatty acid, BHB = β-hydroxybutyrate, TG = triglyceride

**Supplementary Table S3.** Least squares means (LSM) and 95% confidence intervals (CI) of phenotypic responses for cows more (MR) or less resistant (LR) to lipid-related metabolic disorders.[1]

| | MR | | LR | | *P*-value | | |
|---|---|---|---|---|---|---|---|
| **Response**[2] | **LSM** | **95% CI** | **LSM** | **95% CI** | **Cluster** | **Time** | **Cluster×Time** |
| BW, kg | 662.80 | [650.45, 675.27] | 648.60 | [636.62, 660.64] | 0.08 | <0.01 | 0.01 |
| BCS, pts | 3.25 | [2.75, 3.83] | 3.28 | [2.76, 3.88] | 0.91 | <0.01 | 0.21 |
| DMI, kg/d | 21.34 | [19.36, 23.16] | 19.80 | [17.46, 21.90] | 0.23 | <0.01 | 0.21 |
| Milk Energy, Mcal/d | 35.65 | [30.09, 40.45] | 35.66 | [30.11, 40.46] | 0.99 | <0.01 | 0.35 |
| EBAL, Mcal | | | | | | | |
| Prepartum | 12.92 | [10.71, 14.66] | 12.77 | [10.52, 14.54] | 0.89 | 0.95 | 0.10 |
| Postpartum | -7.28 | [-9.57, -4.99] | -11.77 | [-14.06, 9.48] | 0.01 | 0.01 | 0.48 |
| Plasma glucose, mg/dL | 67.73 | [60.84, 74.61] | 62.05 | [55.33, 68.77] | 0.21 | 0.01 | 0.04 |
| Plasma FA, mEq/L | 0.21 | [0.17, 0.25] | 0.25 | [0.20, 0.32] | 0.12 | <0.01 | 0.74 |
| Serum BHB, mmol/L | 0.51 | [0.42, 0.66] | 0.67 | [0.52, 0.95] | 0.10 | <0.01 | 0.88 |
| Liver TG %, DM | 3.28 | [2.65, 4.05] | 4.60 | [3.72, 5.69] | 0.03 | <0.01 | 0.01 |

[1]Cow disposition to lipid-related metabolic disorders was determined based on K-means clustering of cows originally assigned to a ketosis induction protocol (dietary challenge of excess energy prepartum and feed restriction postpartum) based on lipid metabolite concentrations in blood fractions (plasma or serum) and liver tissue (n = 3 cows per cluster).

[2]BW = body weight, BCS = body condition score, DMI = dry matter intake, EBAL = calculated energy balance, FA = fatty acid, BHB = β-hydroxybutyrate, TG = triglyceride
